# Psychological distance and pro-environmental behavior: Insights from wildfire-affected PCT hikers

**DOI:** 10.3389/fpsyg.2025.1481964

**Published:** 2025-07-02

**Authors:** Lindsay B. Miller, Ronald E. Rice

**Affiliations:** Department of Communication, University of California Santa Barbara, Santa Barbara, CA, United States

**Keywords:** psychological distance, construal level theory (CLT), wildfire experiences, climate change perceptions, pro-environmental behavior (PEB), Pacific Crest Trail (PCT), semi-structured interviews, environmental psychology

## Abstract

**Introduction:**

As wildfires and extreme weather events increase in frequency and severity, understanding individuals’ psychological and behavioral responses to these rising climate change impacts is necessary to cultivate pro-environmental behavior (PEB). Based on a theoretical model grounded in construal level theory and the theory of reasoned action, we propose that exposure to wildfires is associated with psychological distance of climate change, climate change and wildfire attitudes, and PEB; that psychological distance is associated with climate change attitudes and PEB; and that subjective norms are associated with PEB.

**Method:**

We assess these associations through an *a priori* content analysis of 66 semi-structured interviews with Pacific Crest Trail hikers during the 2022 wildfire season, illustrated through quotes responding to interview questions asking about such associations.

**Results and discussion:**

The analyses and quotes provide initial support for the proposed model, nuanced insights into the subdimensions of each construct, and a basis for possible wildfire and climate change messaging.

## 1 Introduction

As climate impacts such as extreme wildfires become increasingly salient (e.g., Diffenbaugh et al., 2021), understanding their psychological and behavioral consequences is necessary to cultivate environmental action. However, how extreme weather events such as wildfires may be leveraged to promote pro-environmental behavior (PEB) remains unclear. One barrier to PEB is the common perception that climate change impacts will occur in the future, in distant locations, to other people, and with uncertainty, which reduce individuals’ motivation to personally combat the climate crisis (Griskevicius et al., 2012). In contrast, wildfires are glaringly proximal on all four of these dimensions, especially for those who directly experience them. Construal level theory (CLT) suggests that these dimensions of psychological distance (temporal, spatial, social, and/or hypothetical) inhibit engagement with climate change issues and other PEB (Maiella et al., 2020; Trope and Liberman, 2010). Thus some research (noted below) indicates that reducing psychological distance can increase such behavior, while others argue this approach by itself is insufficient. These mixed results may be the result of both conceptual and methodological issues.

Conceptually, the relationship between psychological distance and PEB is likely complicated by additional influences (Maiella et al., 2020). Scholars have argued that integrating CLT with constructs from the theory of reasoned action (TRA; Fishbein, 1979) and the later theory of planned behavior (TPB; Ajzen, 1991) can better explain engagement in climate-friendly behavior (Brinkerhoff, 2020; Deng et al., 2017; Tulone et al., 2020). In particular, attitudes and subjective norms—the two antecedents of behavior intention in the TRA—have been shown to influence behavior differently depending on psychological distance (Ledgerwood, 2008). Accordingly, this study integrates the original TRA with CLT, as it offers a more parsimonious fit for our aims. Moreover, the TRA provides a flexible foundation for model extensions—a common approach in environmental behavior research. For example, scholars have extended the TRA/TPB with personal goals and motivation (Islam et al., 2024), natural environment, cultural atmosphere, and emotions (Wang et al., 2025), cost and availability of alternatives (Oludoye et al., 2024), and moral norms and trialability (Valizadeh et al., 2023a), and by introducing additional paths between behavioral antecedents and PEB (Valizadeh et al., 2023b). Thus, the TRA provides a theoretically justified set of variables that can be appropriately integrated with CLT constructs.

Methodologically, many existing studies assess psychological distance using experimental manipulations or retrospective self-reports. However, psychological distance is context-specific and dynamic (Brügger, 2020; Trope and Liberman, 2010; Wang et al., 2021); to produce valid insights, data should be collected during or immediately following real-world events. While agent-based modeling and simulation can propose how climate events may relate to PEB (Ribeiro-Rodrigues and Bortoleto, 2024), this study investigates whether and how personal experiences of wildfires relate to psychological distance of climate change, attitudes, subjective norms, and PEB, based on individuals’ lived experiences.

We take a novel approach to the study of psychological distance through developing a model based on CLT and the TRA, examined through content analysis of 66 semi-structured interviews conducted with Pacific Crest Trail (PCT) hikers during peak wildfire season in the summer of 2022, illustrated through participant quotes about the proposed associations. Through this investigation, we aim to (a) shed light on whether and how individuals’ perceptions of climate change are shaped, reinforced, or change as a result of exposure to wildfires and other extreme weather events, and (b) provide insights into effective mechanisms to increase PEB during extreme weather events. As environmental psychologists increasingly prioritize solutions-focused research, this study helps to diversify the discipline’s methodological toolbox and probe deeper into the lived experiences of those exposed to climate change impacts (Nielsen et al., 2021). Understanding whether and how individuals who experience extreme weather events (here, wildfires) are motivated to engage in climate action (here, pro-environmental behaviors) can facilitate the design, dissemination, and evaluation of behavior interventions during times of climatic disruption.

## 2 Literature review and theoretical foundations

### 2.1 Wildfires and climate change

Wildfires and extreme weather events are increasing in frequency and severity, in part due to the drying and heating of atmosphere associated with climate change (Diffenbaugh et al., 2021; Intergovernmental Panel on Climate Change, 2021; MacDonald et al., 2023; see also Valiant, 2023, and Goodell, 2023 for books on global fire and heat). Except for 2022, the last half-dozen years have witnessed the highest number of extreme wildfires on Earth, increasing 220% since 2003; they are also becoming more intense (Cunningham et al., 2024). Since 1970, the average length of wildfire season in the western U.S. has increased by more than 100 days, and the number of acres burned has grown 600% (Environmental Defense Fund, 2024).

Although the ways in which forests are managed can affect fire severity, climate change also plays a central role in the recent growth in regional wildfires. Analyzing data from 1979 to 2020, Jain et al. (2022) show that the fire weather index, initial fire spread index, and vapor pressure deficit have all increased around 12%, influenced by lower relative humidity and increased temperature due climate change. Furthermore, these influences are projected to worsen in coming years (Jain et al., 2022). Climate change also affects the jet stream in ways that can divert rain from vulnerable areas while increased heat reduces snowpack and its water melt (Environmental Defense Fund, 2024), which makes areas more susceptible to wildfire. These contributions of climate change to wildfire patterns lead many environmental researchers to conclude that “extreme weather enhanced by climate change is increasing the duration of the fire season and occurrence of extreme fire weather and events” (Schweizer et al., 2020, p. 41; see also Wasserman and Mueller, 2023).

### 2.2 Exposure to extreme weather events

As climate change intensifies, individuals across the globe are experiencing its impacts. As many as 71% of US residents report having experienced extreme weather in their community in 2022, with 21% experiencing extreme wildfires (Leppert, 2022). A growing literature suggests that individuals who are exposed to these extreme weather events may exhibit higher levels of climate change concern and risk (Lidskog et al., 2019; Spence et al., 2011) and stronger beliefs in climate change (Reser et al., 2012). Experiencing these events has also been associated with behavior change such as disaster preparedness (Dessai and Sims, 2010; Silver and Andrey, 2014; Spialek et al., 2021) and intention to engage in PEB (Rudman et al., 2013; Spialek et al., 2021). Others have found that the relationship between extreme weather experience and PEB was mediated through climate change perception (Deng et al., 2017; Spence et al., 2011). To explain the relationships between direct climate change experiences, environmental attitudes, and pro-environmental behavior, some researchers have turned to construal level theory of psychological distance.

### 2.3 Psychological distance and construal level theory

The concept of psychological distance includes four interrelated dimensions: *temporal distance* represents the distance between now and the object’s point in time; *spatial distance* represents the distance between here and the object’s location; *social distance* represents the difference between “self” and “other” (Smith and Trope, 2006; Trope and Liberman, 2010); and *hypothetical distance* represents “the distinction between real and imagined objects and between probable and improbable events” (Trope and Liberman, 2010, p. 7). Many scholars propose that in the absence of direct experience of its impacts, climate change is likely to be perceived as psychologically distant (affecting other locations, the long term, other people, and with uncertainty), and that this psychological distance can impede environmental action (e.g., van der Linden et al., 2015). Indeed, reducing psychological distance of climate change, whether experimentally or naturally as climate change impacts become more apparent, is frequently suggested as a strategy to mobilize PEB (e.g., Jones, 1989; Schuldt et al., 2016; Spialek et al., 2021; van der Linden et al., 2015). However, there is little evidence for the efficacy of this approach on its own (Brügger et al., 2016; Chen, 2020; McDonald et al., 2015; Schuldt et al., 2018; Spence et al., 2011; Wang et al., 2022, 2021). Researchers also caution that many of the findings that support this claim are correlational, and that the relationship between psychological distance of climate change and PEB does not guarantee that interventions designed to decrease psychological distance will inspire more climate action (Wang et al., 2021).

Construal level theory (CLT) is a frequently used framework for understanding the effects of psychological distance on attitudes and behavior (e.g., Wang et al., 2021). CLT argues that when attitude objects are psychologically distant, individuals form abstract mental representations, or construals, of the object, and will therefore be more influenced by other psychologically distant stimuli (Trope and Liberman, 2010). In contrast, when individuals perceive that an attitude object is psychologically proximate (e.g., they directly experience climate change impacts), they will construe the object concretely, and they will be more influenced by other psychologically proximate and concrete stimuli (Spence et al., 2012; Trope and Liberman, 2010). For example, prior research has found subjective norms (based on one’s immediate social context) are more influential on psychologically proximate attitude objects, and individual attitudes (based on broad principles that apply across situations) are more influential on psychologically distant attitude objects (Ledgerwood, 2008; see also Bijani et al., 2017).

Although CLT provides an important basis for understanding how climate change is mentally represented, Brügger et al. (2016) note that “from the perspective of construal level theory, decreasing psychological distance should not itself influence people’s willingness to act but change the processes that underlie individual decision-making” (p. 125). Therefore, recent research suggests that the relationship between psychological distance and behavior may be strengthened by additional explanatory variables (Brinkerhoff, 2020; Chen, 2020; Jia et al., 2021). To better understand behavior, various scholars have integrated the cognitive constructs of the CLT with constructs from the TRA and the later TPB that relate to behavior intentions and change (Brinkerhoff, 2020; Deng et al., 2017; Tulone et al., 2020).

### 2.4 The theory of reasoned action

Fishbein’s (1979) theory of reasoned action suggests that a person’s behavior is best predicted by their predisposition toward the action, which itself is predicted by both subjective norms and one’s attitudes. Subjective norms refer to the perceived social pressure from important others to perform (or not perform) a behavior (Fishbein, 1979). Environmental attitudes are commonly viewed as stable evaluative tendencies that reflect individuals’ environmental concern and values (Miller et al., 2022), and influence people’s beliefs about, affect toward, and behavioral responses toward the environment (Milfont and Duckitt, 2010). Given CLT findings that subjective norms are more influential than individual attitudes when attitude objects are psychologically proximate (Ledgerwood, 2008), and that environmental attitudes are affected by perceived susceptibility to climate change impacts (Shen et al., 2024), integrating the TRA with CLT may clarify diverging findings in the literature by exploring the constructs that enable or constrain the relationship between psychological distance of climate change and PEB. Following CLT’s tenet that stimuli are more strongly related when matched with construal level, this study examines both *climate change attitudes* (more abstract) and *wildfire attitudes* (more concrete).

The original conceptual framework of the TRA specifies that attitudes and subjective norms should each be measured in relation to a specific target behavior, and examined through measures of the beliefs underlying these constructs (Fishbein, 1979). Accordingly, studies based on the TRA “should normally consist of two parts: qualitative (identification of antecedent beliefs) and quantitative (statistical assessment of direct and indirect variables)” (Yuriev et al., 2020). Although many examinations of PEB through the TRA and TPB relate antecedents to a specific target behavior, far fewer examine the underlying attitude and subjective norm beliefs (Yuriev et al., 2020). Further, meta-analyses on TPB antecedents have found that general attitude and norm measures still explain significant variance in PEB (Geiger et al., 2019).

### 2.5 Pro-environmental behaviors

Pro-environmental behavior is “behavior that consciously seeks to minimize the negative impact of one’s actions on the natural and built world” (Kollmuss and Agyeman, 2002, p. 240). Previous research on experiences of extreme weather events suggests that exposure can lead to behavior change to guard against future similar events (more concrete; Deng et al., 2017; Silver and Andrey, 2014; Spialek et al., 2021), but that it can also affect PEB in domains unrelated to the extreme weather (more abstract; López-Feldman and González, 2022; Reser et al., 2012; Spence et al., 2011). Therefore, in the context of extreme weather events, we distinguish between PEBs that relate to the event itself (*wildfire PEB;* behaviors that relate to helping to prevent wildfires) and other PEB (*general PEB;* behaviors related to more general environmental contexts). These two categories represent individuals’ general propensities to engage in (general or wildfire-specific) environmental action (Lange, 2024).

## 3 Model and hypotheses

**Figure 1:**
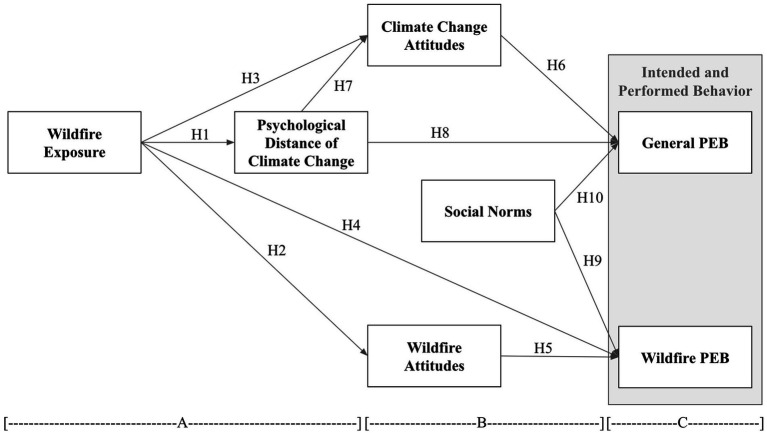
Main relationships. Constructs in A are from construal level theory; constructs in B are from the theory of reasoned action; constructs in C are from both.

Based on the above review, Figure 1 presents the overall model of the main associations, indicating the corresponding hypotheses. The model proposes that wildfire exposure is associated with H1 psychological distance of climate change, H2 wildfire attitudes, H3 climate change attitudes, and H4 wildfire PEB. In turn, psychological distance of climate change is associated with H7 climate change attitudes and H8 general PEB. Climate change attitudes H6 are associated with general PEB, and H5 wildfire attitudes are associated with wildfire PEB. Finally, social norms influence H9 wildfire PEB and H10 general PEB. We state these associations as hypotheses, because they are justified by the literature and theoretical foundations, but these are not statistically tested. Rather, they are assessed by the presence of the *a priori* content codes in the comments; i.e., the associations are not tested in a causal research design but are based on participants’ comments about those associations.

## 4 Method

We content analyzed the qualitative interview data using an a priori and reliable coding scheme (Table 1), an approach used in prior studies (e.g., Baxter and Eyles, 1999; Kleinheksel et al., 2020; Neuendorf, 2019; Weston et al., 2001). This allows us to provide thick descriptions to understand the nuances of the model that are nonetheless firmly grounded and validated in the literature and the overall model. Thus, we do not seek, report, or analyze emergent or inductive codes or themes, nor use the coded content to generate grounded theory.

**Table 1:** Operationalization of constructs from the theory of reasoned action and construal level theory.

Theoretical framework	Construct	Operationalization via codes
Construal Level Theory	Concrete Experiences/Salience of Wildfire	Descriptions of (separate codes): Walking through burn areas Walking through smoke Smelling or seeing smoke at a distance Seeing ash Avoiding previously burned area* Leaving the trail or rerouting because of active fire* Evacuating due to immediate danger from active fire*
	Reductions in Psychological Distance	Reduced distance due to wildfire exposure Temporal Spatial Social Hypothetical
	Psychological Impact	Negative Psychological Reaction *to wildfire exposure*
	Wildfire Exposure Impact on PEB	Descriptions of wildfire exposure experiences shaping, changing, or reinforcing (separate codes): Wildfire-specific PEB General PEB
Theory of Reasoned Action	Attitudes	Change in or reinforcement of (separate codes): Wildfire Attitudes Climate Change Attitudes
	Subjective Norm Influence on PEB	Social Influence on PEB, including from (separate codes): PCT hikers Friends/family Trail locals Media Each separated into influence on (separate codes): Wildfire PEB General PEB

*Experience involved behavior change.

### 4.1 Site

**Figure 2:**
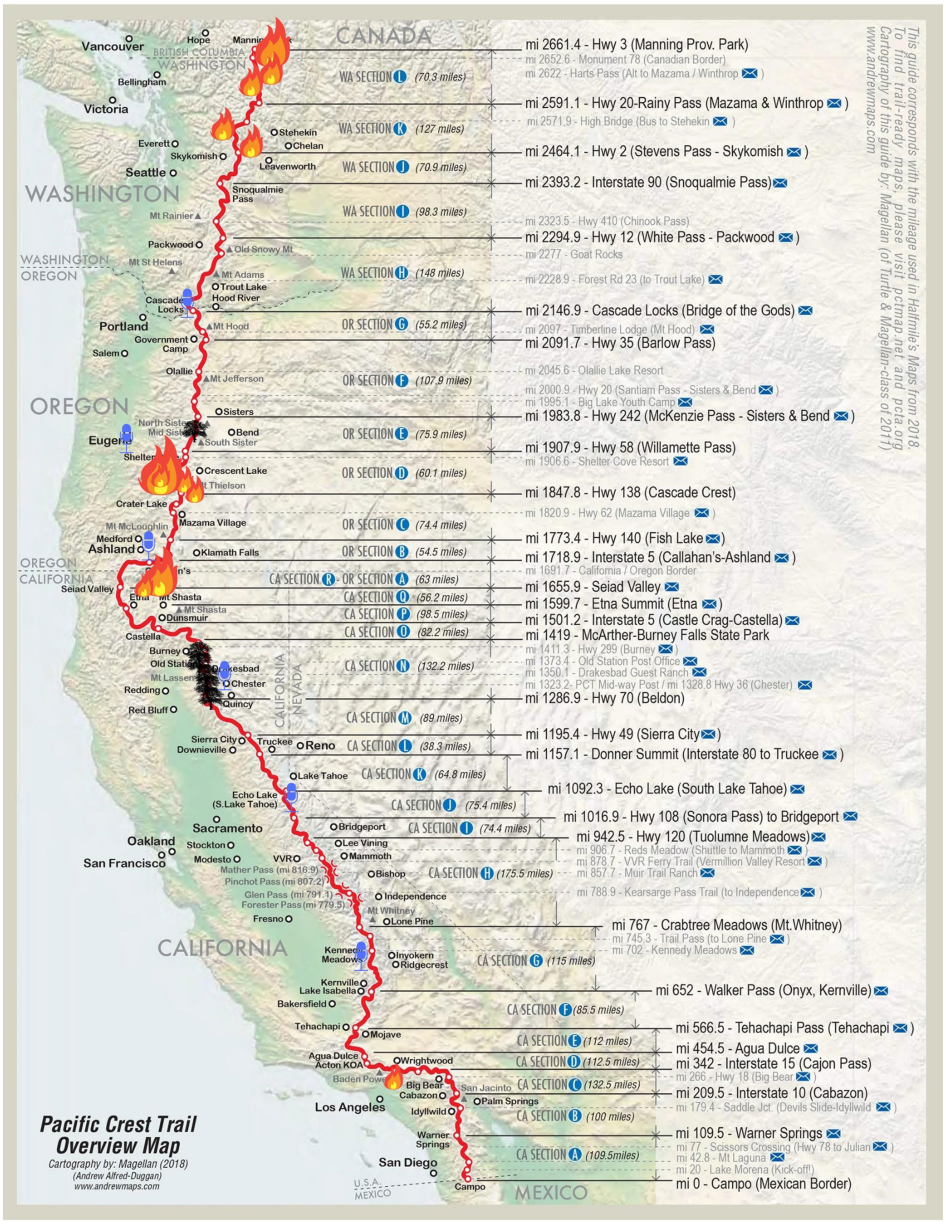
Map of the Pacific Crest Trail, including wildfires, previously burned areas, and interview locations along the PCT. Underlying map source: www.pcta.org. Sections indicate PCT markers. The flame symbol indicates an active fire at the time of the interviews, black trees indicate large previously burned areas, and microphone icons indicate the interview locations.

The Pacific Crest Trail (PCT; see Figure 2) was selected as a site due to the annual disruption of wildfire. Each year, over 4,000 people receive permits to hike the entire PCT from Mexico to Canada during peak fire season in the Northwest. Each year, sections of the PCT close as wildfires burn across or near the trail (Gerety and Trinca, 2022; Harrell, 2021). For long-distance PCT hikers who spend months preparing to walk the 2,652 miles, the presence of an active wildfire creates physical threats and disruptions, along with emotional disruptions due to the incineration of the trail which has become their home, and the inability to complete this long-sought goal. Feedback on study materials was obtained from a pretest sample of undergraduates and from the Pacific Crest Trail Association, generating a final structured interview guide. The project was publicized on Facebook and Instagram PCT accounts, and on posters and fliers that were delivered and mailed to PCT resupply towns and campsites popular during the early months of the season.

### 4.2 Positionality

The first author registered for and hiked the PCT between April–July 2022, hiking over 1,200 miles from the Southern Terminus to Quincy, CA. This included walking and camping with other PCT hikers, being given a trail name, and importantly, being affected by environmental obstacles, wildfire smoke, prior wildfire burn areas, and active fires. Despite designing this project with the intention of studying wildfire impacts, the first author was surprised by the profundity of the physical and emotional impacts of the wildfires when they emerged, both personally and as noted by interviewees. Experiencing the tragedy of having a section of the trail and a yearlong plan go up in smoke overnight lent an understanding of the context of participants’ comments and credibility to the results.

### 4.3 Sample

The first author conducted semi-structured interviews with hikers willing to participate in the interview while in resupply trail towns. Thus the researcher engaged with individuals who were guaranteed exposure to different levels of wildfire: they saw flames and ash; smelled and saw smoke and haze; were evacuated; had their plans disrupted; walked through burned areas; and communicated with other hikers about their experiences. This method allowed for the collection of rich data from individuals who were experiencing different levels of wildfire in real time.

### 4.4 Semi-structured on-site interviews

Corresponding to the recommendation noted earlier that CLT data should be collected during or immediately following real-world events, conducting interviews in the context of the PCT—where hikers are more likely to have been or would be exposed to wildfires—both increases the relevance of their comments to CLT and helps to better understand their lived experiences. The semi-structured interview guide (see Table 2, which also provides rationales for each set of questions) represented the project motivations and theoretical foundations of the model. The interviews followed the semi-structured guide, while maintaining flexibility for participants to tell their stories. Questions that had already been addressed in the participant’s prior responses were not asked, and probes were only asked as needed.

**Table 2:** Semi-structured interview guide

**#**	**Interview questions**	**Probes**
**1**	Tell me to what extent you have personally experienced aspects of wildfires.	1a: Can you give me an example of a time when you experienced an aspect of wildfire? 1b: Can you explain a little about the effects of that experience on you?
**2**	Do you believe that your experiences of wildfires influence the way you think about climate change? Explain.	2a: Do you view wildfires as a current consequence of climate change? Explain. 2b: In what ways do your experiences of wildfires change how near you feel to the effects of climate change? 2c: In what ways do your experiences of wildfires change how soon you believe the effects of climate change will occur? 2d: In what ways do your experiences of wildfires change who you expect will experience the effects of climate change? 2e: In what ways do your experiences of wildfires change how certain you are that climate change is occurring?
**3**	In what ways are your experiences of wildfires influenced by other people?	3a: Who are the people who are relevant to your experiences of wildfires? 3b: What are the influences of people who are relevant to you?
**4**	What types of social situations influence the way you think about wildfires?	
**5**	To what extent do you feel your engagement in behaviors that are beneficial to the environment is influenced by other people?	5a: What are the influences of people who are relevant to you? 5b: Who are the people who are relevant to your engagement in environmentally beneficial behaviors?
**6**	What types of social situations influence the kinds of environmental behaviors you engage in?	
**7**	To what extent do you feel that your experiences with wildfires influence your intention to act in ways that are beneficial to the environment?	7a: What types of behaviors do you think are most influenced by your experience of wildfires?

Questions 5, 6, and 7 specifically refer to PEB; however, participants may refer to PEB in responding to the other questions. The unit of coding analysis was the complete set of responses to all the questions, for each interviewee.

Sixty-seven interviews were conducted, though one was cut short, resulting in 66. The interviews were conducted in limited number throughout the beginning of the hiking season, and in concentration once wildfires along the PCT began at the end of July 2022. The PCT Map in Figure 2 shows then-active wildfires, burned areas from prior fires, and the interview locations. Respondents were mostly from the United States (US) (46), but some from other countries: Germany (5), Canada (3), Australia, Denmark, England, and the Netherlands (2 each), and one each from Slovenia and Sweden. Sixteen respondents were female, 45 were male, and one respondent identified as non-binary (note that four interviews were conducted with two respondents). Upon completion of the study, the anonymized audio files were transcribed using a professional service.

### 4.5 Content analysis

Content analysis may be qualitative (thematic) or quantitative (frequency of codes), and *a priori* (reflecting a developed model or explicit baselines) or emergent/inductive (allowing for codes and subsequent themes to emerge from the data and researcher interpretation; Neuendorf, 2017). Here, we conduct quantitative a priori coding. To analyze the comments, we developed an initial a priori coding scheme spreadsheet reflecting the motivations and theory of the study, as reflected in Figure 1, as well as the wording and sequence of the interview guide. Table 3 lists the main categories and specific codes. The main categories correspond to the concepts in the Figure 1 model; the specific codes were identified by the authors through close reading of the comments as specific instances or forms of the main categories. The coding unit was each entire interview. Two authors and two trained assistants coded the transcripts by first reading the entire interview. Then they returned to the beginning and started coding (entering the transcript line number) following the spreadsheet. However, the two final attitude entries were coded after initial coding and again revisiting the entire interview. All four members coded and discussed questions, ambiguities, or suggestions for improved operationalization through multiple initial sets of five interviews each until we achieved basic stability of the codebook. Early difficulties with several codes and interview ambiguities resulted in dropping these codes. The first author and the two assistants then proceeded with production coding.

**Table 3:** Content analysis codes and descriptive statistics.

Code	*N*	*M*	SD
Interview record	66		
Interviewee: miles hiked		1587.6	527.7
Interview transcript word count		3,300	1279.6
Interview duration (minutes)		22.9	8.2
Wildfire exposure			
Observed		2.06	0.99
Walked through a previously burned area	58	0.88	0.33
Walked through PCT wildfire smoke	25	0.38	0.49
Saw or smelled smoke	43	0.65	0.48
Saw ash	10	0.15	0.36
Acted		1.15	0.83
Rerouted to avoid a previous burn	16	0.24	0.43
Did not hike or avoided a section because active wildfire-related issue	47	0.71	0.46
Evacuated from PCT because of immediate threat of wildfire	13	0.20	0.40
Other			
Intensity of response: grouped into none or any	52	0.79	0.41
Wildfire experiences prior to PCT	26	0.39	0.49
Psychological distance (Decrease in distance of climate change associated with wildfire experience)		1.95	1.40
Temporal	33	0.50	0.50
Spatial	27	0.41	0.50
Social	35	0.53	0.50
Hypothetical	34	0.52	0.50
Influence of social norms: topic and source			
On general pro-environmental behaviors (source)		1.35	0.98
PCT hikers	38	0.58	0.50
Friends/family not on the PCT	28	0.42	0.50
Individuals local to the PCT or officials	8	0.12	0.33
Media	15	0.23	0.42
On wildfire pro-environmental behaviors (source)		0.12	0.37
PCT hikers	5	0.08	0.27
Friends/family not on the PCT	1	0.02	0.12
Individuals local to the PCT or officials	1	0.02	0.12
Media	1	0.02	0.12
Attitudes (reinforcement or change)		1.42	0.77
Wildfire	49	0.74	0.44
Climate Change	45	0.68	0.47
Influence of wildfire exposure		0.85	0.68
On general pro-environmental behaviors (examples: unrelated to minimizing wildfire; e.g., take train instead of flying; get involved with an environmental organization)	31	0.47	0.50
On wildfire pro-environmental behaviors (examples: cleared flammable debris away from camp stove before lighting it; said something to people whose behaviors could risk starting a wildfire)	25	0.38	0.49

Values = number of respondents mentioning that coded content, out of *N* = 66. Except for miles hiked, means for each code are the percentage of respondents mentioning this coded content in their comments. Values for the header variables are the total percentages combined across their constituent codes. Full codebook available from the authors.

To assess inter-coder agreement, all coded line numbers were converted to 1 s, with 0 indicating no code. The values for each coder for each code were calculated using Freelon’s ReCal3 (Freelon, n.d.). However, Cohen’s Kappa and Krippendorff’s Alpha produced some nonsensical results due to the low variance in many codings. This is a common phenomenon; Feng (2015) and Zhao et al. (2018) discussed these issues, with the latter especially emphasizing the validity of using agreement percentages for such contexts. Therefore we used the percent agreement across the three coders or across pairs of coders to assess progress toward agreement levels, by coders, by individual codes, and across all codes. Joint coding among the first author and two assistants was formally compared for 11 sets of interviews, and the two assistants independently coded three sets each. The average final unweighted agreement across all 29 joint codings was 85%, with a median of 100%. Remaining disagreements for joint coding were resolved by majority coding.

## 5 Results

Table 3 presents the descriptive statistics. The interview questions and thus the content analysis codes often are about associations between two variables (e.g., whether wildfire experiences influenced participants’ perceptions of climate change), so most responses describe perceived causal relationships. Table 4 provides illustrative quotes corresponding to the subsections below, to support the validity of the results.

**Table 4:** Illustrative quotes for content-analyzed relationships.

Results section	Illustrative quotes
5.1 Wildfire exposure	
Hikers noted the psychological impact of seeing their temporary “home” now threatened or destroyed. Despite the lesser threat to hikers’ immediate safety, the 106-mile stretch of forest that burned in 2021 (the “Dixie Fire”) was often described by participants as one of their most impressionable experiences.	“Eventually, hopefully if you really let it, this trail becomes your home. You do not have another home. Most people have given up their houses and leases and jobs, and everything. This is your home, and your home’s on fire. It’s not just on fire for a day. Like hundreds of miles of your home are gone overnight. There’s no way that’s not traumatic in a way that I do not think most people can understand” (20). “It’s really bleak. It’s really dusty. The views are not awesome. Everything’s just black. It’s dangerous because there is going to be a lot of trees that you cannot sleep under. It’s very challenging. Also, we did not wanna skip a mile, so we went through that entire section just to be stopped by a new wildfire. You can understand how that would be such a morale breaker” (20).
5.1.1 Association between wildfire exposure and psychological distance of climate change (H1)	
Description of reduction in *hypothetical distance* of climate change due to wildfire exposure Description of reduction in *social distance* of climate change due to wildfire exposure Description of reduction in *temporal distance* of climate change due to wildfire exposure Description of reduction in *hypothetical distance* of climate change due to wildfire exposure	“It’s a sh**ty thing where you only realize something’s going on when it hits you or when you personally experience it. Before that you close your eyes and do not really look. That really changed it for me. I’ve heard about California fires since forever, because that’s in the news, but you are like, ‘Oh, that’s horrible’ but then you do something else. That was really eye-opening” (35). “It’s hard to take something as large-scale as climate change and then see how it might immediately and directly impact you. Then walking through a really severely burnt area, you are directly being impacted by it walking through… It’s a good reminder of how direct the effects of climate change can be on your life” (50). “I remember my whole upbringing has always been like, not in your lifetime, but in your children or grandchildren’s lifetime, and we gotta change things now for the other generations to come, but it’s very much feeling like our generation now and not so much about the future” (52). “the wildfires this year has been sort of driving home that it is happening here. It is around us. It’s something that we are gonna have to deal with” (63).
5.1.2 Association between wildfire exposure and attitudes	
5.1.2.1 Wildfire Attitudes (H2)	
Hikers described increased pessimism about the state of wilderness areas after experiencing wildfire impacts on the PCT. Some hikers explained that hiking through previously burned areas also brought them a sense of optimism and beauty.	“Being there [in the Dixie Fire burn area] in the moment really cemented how catastrophic these modern fires are, and honestly kinda gave me a bit of a grim outlook for the future. I do not know if I’m gonna be able to continue to recreate in the forests for the rest of my life, or if it’s just gonna all be charred by the time I’m old. If I have kids, are they gonna be able to see the healthy mixed forest that we get to hike through, or is it just gonna be gone?” (63) “…you also get to see what rebirth looks like. I’ve walked through a forest that was completely charred, but I saw probably hundreds of thousands of mushrooms all over the trees. You’re seeing life come up from the ground, and you are like, ‘Man, this is beautiful.’” (2)
5.1.2.2 Climate change attitudes (H3)	
Many hikers explained that while their beliefs had not changed per se, those were reinforced, confirmed, or intensified after their experiences with wildfires. Not all hikers attributed the severity of the wildfires to climate change, with some pointing to forest management as the culprit, and others describing the complex nature of climate change impacts tied up with fire suppression tactics.	[The wildfires had a] “huge influence on my attitude toward climate change. I wasn’t anti-climate change, not a skeptic or anything like that, but it’s just reinforced my beliefs even more so on the trail” (21). “out here, I see the fires more as a management problem. Of course, there were always fires here, but with the suppression tactics to suppress the fires for 50 years and build up a huge pile of biomass… of course, at some point, it starts. Then you cannot control it anymore” (53).
5.1.3.2 Association between wildfire exposure and wildfire PEB (H4)	
Some hikers described being motivated to engage in wildfire PEB because of their perceived increase in the risk of wildfires or of unsafe fire behaviors. Some participants described feeling hopeless to prevent future wildfires.	“When you have lived through it, experienced it, you realize that you cannot mess around. You’ve got to be really strong on your behaviors and follow all the guidelines and pull people up if they are doing risky behaviors that create a fire hazard or fire risk” (46). “Waste free, I’m like oh, yeah, I can just use less things, but a wildfire I’m like how do I fit into that model of help to stop it from happening?” (22)
5.2 Attitudes as a predictor (H5, H6)	
A hiker described how his newfound awareness of climate change motivated him to educate others about general PEB.	“That’s something that I want to carry forward is to keep people aware of what’s happening, in a sense which was not how I was before. I was just oblivious of what was happening. I think that’s the one where it had changed for me after-trail” (24).
5.3 Psychological distance of climate change as a predictor	
5.3.1 Associations with climate change attitudes (H7)	
Participants described how the proximity of climate change, brought on by their exposure to wildfires, increased their concern for and awareness of general environmental issues. Some hikers also felt that it was easy to have an ambivalent attitude until confronted with climate change impacts.	“It makes it in the forefront. It makes really immediate. It makes is so that you cannot ignore what’s happening… The fires and those other elements made me begin to really focus on what is actually happening; what is being predicted; the models; and just facing that. Like facing that hard truth head on” (20). “I think when the fire’s at our front doors, is threatening our own properties and our own livelihoods, we might focus on it more. Until then, a fair level of nonchalantness will be somewhat acceptable” (65).
5.3.2 Associations with PEB (H8)	
Some hikers described being motivated to engage in PEB based on the perceived proximity of climate change. Some hikers felt that certain dimensions of psychological distance (in this quote, social distance) motivated them to engage in PEB. Some hikers described increasing apathy and hopelessness in response to the psychological proximity, and a concern that their actions would not continue after the wildfire was no longer top of mind.	Referring to climate change: “I’ve been taught it, I’ve understood it, I believe it, thankfully. Just to see it firsthand is another level. It makes me want to be more active in the change to make climate change better, if possible” (32). “when those experiences or threats become impactful for those people surrounded, that to me is a catalyst for change and for action that I do not even have to think about. It’s just something you do” (4). “I think it makes me want to do things, but realistically wanting to make changes and actually making the changes is a huge step” (47).
5.4 Association of subjective norms with PEB (H9-H10)	
Some hikers described how spending time with outdoors enthusiasts gave them new motivation to perform (especially general) PEB. Many hikers described how observing people engaging in PEB was more motivational than being publicly shamed for inaction. However, for hikers who were more environmentally conscious at home, subjective norms on the trail had a negative effect on their engagement in PEB.	“I think the leading by example is really key, and I feel like out here, we are lucky enough to hang out with a bunch of people who really care about the environment” (48). “If a group of people has similar values and they act a way without calling attention to it, if three outta four people are separating their trash but not making a thing out of it, then I think that the fourth person a lotta times it makes it easier for them to just follow suit instead of being told or taught” (13). “I’m vegan, I only ride a bike. I do not have a car… If at home I would come to a party with M&Ms. and Skittles, people would be like, ‘What the f**k are you doing?’ I would get harsh judgement for it. Here it’s the norm. That of course influences me. Because I do think we need other people to keep us in check, to regulate us” (35).

### 5.1 Wildfire exposure

In total, the 66 participants mentioned 136 instances of observing and/or experiencing wildfire impacts and 154 instances of acting in response to wildfires. Participants experienced a wide range of wildfire impacts: some noted only having experienced previous wildfires when walking through burned forests, while some evacuated from the trail due to immediate wildfire danger. However, hiking through a previously burned area was the most prevalent mention of observations/experiences (*n* = 58), and avoiding a section of trail due to an active wildfire was the most common behavioral response to wildfires (*n* = 47). Across the diverse wildfire experiences (both those that involved observing and acting in response to wildfires), hikers noted the psychological impact of seeing their temporary “home” (20) now threatened or destroyed. Despite the lesser threat to hikers’ immediate safety, this psychological impact was frequently associated with the 106-mile stretch of forest that burned in 2021 (the “Dixie Fire”), described by one as “really bleak… a morale breaker” (20). Thus, experience observing the impacts of previous wildfires was often equally, or more, poignant to participants as was evading threats of active wildfires.

#### 5.1.1 Association between wildfire exposure and psychological distance of climate change (H1)

Most participants (*n* = 55) mentioned their experiences with wildfires affecting one or more of the four dimensions of psychological distance. Participants who did not hike or avoided a section of the trail because of an active wildfire-related issue (e.g., smoke or flames) were more likely to perceive climate change as psychologically proximate because of their wildfire experience.

The four dimensions of psychological distance were all represented about equally across the interviews (27, 33, 35, and 34 instances for spatial, temporal, social, and hypothetical, respectively, for a total of 129). Although most participants believed in climate change and were aware of wildfire impacts through the media, they expressed reductions in *hypothetical distance* through descriptions of their experiences being “eye-opening” as to the reality of climate change (35). *Social distance* was emphasized through the recurring theme of perceiving climate change affecting hikers personally, or affecting people close to them, with some feeling that “it’s a good reminder of how direct the effects of climate change can be on your life” (50). Reductions in *temporal distance* were represented through descriptions of climate change feeling like a problem for “our generation now and not so much about the future” (52). Finally, reductions in *spatial distance* were described by some hikers who no longer felt like climate change only affected other parts of the world, and they could now see that it was “happening here” (63). Thus, across all four dimensions of psychological distance, participants generally perceived climate change as being closer due to their wildfire experiences.

#### 5.1.2 Association between wildfire exposure and attitudes (H2, H3)

##### 5.1.2.1 Wildfire attitudes (H2)

Forty-nine participants described an increase in, reinforcement of, or confirmation of their concern about wildfires, their perception of the seriousness of the problem of wildfires, their worry about wildfires, and/or their general perspective about wildfires. Both hiking through a previously burned area and negative psychological reactions to the wildfires were associated with wildfire attitudes in some of the comments.

Hikers described increased pessimism about the state of wilderness areas after experiencing wildfire impacts on the PCT, with one describing that they “gave me a bit of a grim outlook for the future” (63). Often, they expressed concern that wildfires could make the PCT impossible to hike in just 5 or 10 years, or would prevent future generations from being able to enjoy the outdoors. Not all hikers expressed only pessimistic wildfire attitudes as a result of their experiences, however. Some hikers also explained that hiking through previously burned areas brought them a sense of optimism and beauty, especially at observing the regrowth of the forest—a phenomenon also observed by Lidskog et al. (2019).

##### 5.1.2.2 Climate change attitudes (H3)

Forty-five participants described an increase in, reinforcement of, or confirmation of their concern about climate change, their perception of the seriousness of the problem of climate change, their worry about climate change, and and/or their general perspective about climate change. In some comments, climate change attitudes were associated with hiking through a previously burned area and with negative psychological reactions to the wildfires, similar to those for wildfire attitudes.

Most hikers emphasized that they already believed in climate change (only one hiker stated disbelief), so they often first expressed that their experiences of wildfires did not change their attitudes. However, after probing deeper, many hikers explained that while their beliefs had not changed per se, those were reinforced, confirmed, or intensified after their experiences with wildfires.

Not all hikers attributed the severity of the wildfires to climate change, however. Several hikers pointed to poor forest management as the culprit of increasing wildfires, or described the complex nature of climate change impacts tied up with fire suppression tactics (e.g., 53, 62, 65). The US Forest Service policy of near-total fire suppression (although recently engaging in more controlled burns and underbrush removal), contributed to the build-up of fire fuel, increasing intensity and spread (Busenberg, 2004; Kolbert, 2024). California, a state the PCT transects, is an illustrative case where such policies have contributed to “an increase in large catastrophic fires not typical of these ecosystems” (Busenberg, 2004, p. 41; see especially Schweizer et al., 2020). Therefore, some hikers explained that while the wildfires may be exacerbated by climate change, it was challenging to determine whether, or how much, each wildfire exposure was due to climate change or to improper human intervention.

#### 5.1.3 Associations of wildfire exposure and pro-environmental behavior (H4, H5, H6)

##### 5.1.3.1 General PEB

While the model does not hypothesize a direct association between wildfire exposure and general PEB, the interview guide allowed participants to describe their own interpretation of the ways in which wildfires affected their PEB. Thus, 31 hikers expressed that experiencing wildfires on the PCT affected their current or future engagement in PEB unrelated to wildfires (general PEB). The behaviors hikers described ranged from recycling, driving energy-efficient vehicles or taking alternative modes of transportation, communicating environmental risks to friends and family, and contacting politicians and voting for environmental policies, among others.

##### 5.1.3.2 Wildfire PEB (H4)

Twenty-five hikers expressed that experiencing wildfires on the PCT affected their current (reported) or future (intended) wildfire PEB (e.g., stove safety). Some hikers described being motivated to engage in wildfire PEB because of their perceived increase in the risk of wildfires or of unsafe fire behaviors, such as one hiker who expressed that “you realize that you cannot mess around” (46).

However, other hikers noted that experiencing aspects of the wildfires did not motivate wildfire PEB. Because the most disruptive active wildfires during the 2022 PCT season were not started by human action, it is possible that participants’ exposure to wildfire underscored how little one’s personal PEB can do to alleviate or respond to this multifaceted problem. It may also be difficult for participants to increase their wildfire PEB, which are limited and more likely to experience a ceiling effect.

### 5.2 Attitudes as a predictor (H5, H6)

The hypothesized model suggested that wildfire attitudes should be associated with wildfire PEB (H5), and that climate change attitudes should be associated with general PEB (H6). However, comments indicated that both attitudes (wildfire and climate change) were associated with general PEB, but not with wildfire PEB specifically. These results align with the lack of support for H4 (that wildfire exposure would be associated with wildfire PEB); see above for potential explanations for this pattern of results.

### 5.3 Psychological distance of climate change as a predictor (H7, H8)

#### 5.3.1 Associations with climate change attitudes (H7)

Supporting H7, each of the four indicators of psychological distance was associated with climate change attitudes in participant comments. Participants described how the proximity of climate change, brought on by their exposure to wildfires, increased their concern for and awareness of general environmental issues. One participant explained that the immediacy of the wildfires “made me begin to really focus on what is actually happening…facing that hard truth head on” (20). Some hikers also felt that it was easy to have an ambivalent attitude until confronted with climate change impacts “at our front doors” (65).

#### 5.3.2 Associations with PEB (H8)

Some hikers described being motivated to engage in PEB based on the perceived proximity of climate change. Referring to climate change, one hiker described how “Just to see it firsthand is another level. It makes me want to be more active in the change to make climate change better” (32). Some hikers felt that certain dimensions of psychological distance motivated them to engage in PEB. For example, one hiker explained that “when those experiences or threats become impactful for those people surrounded, that to me is a catalyst for change and for action” (4). In this case, the participant was motivated to engage in PEB due to the decreasing social distance of climate change impacts.

However, as mentioned previously, other hikers described increasing apathy and hopelessness in response to the psychological proximity, and a concern that their actions would not continue after the wildfire was no longer top of mind. For example, one hiker stated, “I think it makes me want to do things, but realistically wanting to make changes and actually making the changes is a huge step” (47). Some hikers also expressed that they were already doing all they could to support the environment.

### 5.4 Association of subjective norms with PEB (H9-H10)

The interview question asked participants to describe how social influence affected their engagement in PEBs, without specifying whether those were wildfire-related or general, though those were distinguished via coding. The comments were also coded for the four possible source(s) (other PCT hikers, friends and family not on the PCT, individuals who live nearby the PCT, and the media) of that influence.

Over half of the participants (*n* = 38) said that social influence, in the form of subjective norms (especially those from other PCT hikers and individuals local to the PCT), affected their intention to engage in *general* PEB. Hikers often reported being influenced by multiple sources of those norms. However, few specifically mentioned other people as having much influence on participants’ *wildfire* PEB (*n* = 7).

Some hikers described how they had come from a community that was not very environmentally conscious or aware, so spending time with outdoors enthusiasts gave them new motivation to perform (especially general) PEB. These hikers frequently described PCT hikers as “people who really care about the environment” (48). When asked about what kind of norms had the most influence on their PEB, many hikers described how observing people engaging in PEB was more motivational than being publicly shamed for inaction. These findings align with research suggesting that descriptive norms are more powerful behavioral motivators than are injunctive norms (Niemiec et al., 2020). However, some people who were environmentally conscious at home perceived PCT hikers to be less environmentally inclined than their usual social circles. For these participants, subjective norms on the trail had a negative (boomerang; Miller, 2025) effect on their engagement in PEB.

## 6 Discussion

This study provides specific experienced examples of, and general support for, most of the relationships in the theoretical model (Figure 1). For PCT hikers, many felt that their exposure to wildfire was related to decreasing psychological distance of climate change and to both wildfire and general PEB. In turn, psychological distance of climate change was related to attitudes (climate change and wildfire), and some participants mentioned decreasing psychological distance affecting their PEB. Further, participants described subjective norms from various sources influencing their intended and enacted PEB. These findings have implications for theorizing on CLT and the TRA, individuals exposed to extreme weather, and the communication surrounding salient climate change events.

### 6.1 Theory implications

First, these results contribute to research on extreme weather and the psychological distance of climate change (e.g., Jones, 1989; Schuldt et al., 2016; Spialek et al., 2021; van der Linden et al., 2015) by indicating that natural reductions in psychological distance to climate change may correspond with increases in attitudes without meaningful changes to PEB. Many hikers expressed increasing levels of climate change and wildfire concern, risk, and belief, but stated that their behaviors are unlikely to change as a result. However, the results also indicate that exposure to wildfires may not have to relate to psychological distance of climate change to affect wildfire attitudes and PEB, which supports previous research demonstrating that exposure to extreme weather is directly associated with higher levels of climate change concern, risk, and belief (Lidskog et al., 2019; Reser et al., 2012; Spence et al., 2011), disaster preparedness (Dessai and Sims, 2010; Silver and Andrey, 2014; Spialek et al., 2021) and intention to engage in PEB. Therefore, the two direct relationships between wildfire exposure and psychological distance of climate change, and between wildfire exposure and PEB, may be more important than the indirect relationship of wildfire exposure on PEB through psychological distance (though some did indicate this pattern). If findings from PCT hikers reflect psychological processes of other individuals who are exposed to climate change impacts, the global increase in extreme weather events may simultaneously promote the perception that climate change is psychologically closer and also a stronger propensity to engage in environmental action.

Importantly, these findings reflect a growing awareness of climate change as a result of direct exposure to climate change impacts. The qualitative exploration of these associations compliments previous quantitative assessments by demonstrating that some individuals who are exposed to wildfires are aware of the impacts these events have on their perception of climate change, and on their intended and enacted behavior. Construal levels and corresponding psychological distance can be primed without participant awareness (Trope and Liberman, 2010), so participants’ abilities to verbalize these changes in the context of a semi-structured interview is a noteworthy contribution, and one that merits further investigation. Although awareness by itself is rarely sufficient to motivate environmental action (Bergquist et al., 2023), the increased salience of climate change through exposure to extreme weather may be an important precursor to PEB (Spence et al., 2011).

The pattern of relationships between exposure to wildfires, psychological distance, attitudes, subjective norms, and behavior, as indicated by the interviewees’ comments, also highlights the utility of integrating CLT with the TRA (Brinkerhoff, 2020; Deng et al., 2017; Tulone et al., 2020). Many scholars find it useful to extend the TRA and TPB to improve their predictive power (Yuriev et al., 2020). Until recently, however, the integration of exposure to extreme weather events or wildfires and psychological distance with these models has been mostly unexplored, and thus is a central contribution of this study. As climate change impacts become more apparent (Intergovernmental Panel on Climate Change, 2021), understanding how they relate to these central theories and variables in the literature will become increasingly important.

Beyond providing some general but nuanced support of the theoretical model, the coded content and qualitative quotes illuminate many aspects in the study of exposure to extreme weather events, psychological distance of climate change, and PEB. First, despite participants describing a wide range of wildfire impacts (non-behavioral and behavioral), some wildfire experiences, such as hiking through extensively burned areas, were described as being particularly influential for outcome variables. It is likely that different indicators of extreme weather may differentially affect perceived psychological distance, attitudes, and behavior, as suggested in the literature. Indeed, CLT predicts that concrete representations (such as wildfire) can be abstracted in multiple ways (such as climate change, improper forest management, dangerous landscape, etc.), according to one’s goals (Trope and Liberman, 2010). It may be the case that certain wildfire experiences are more frequently construed as climate change impacts, or as motivational for general pro-environmental behavior change. Given that individuals both on and off the PCT are more likely to experience certain wildfire impacts than others (e.g., seeing or smelling smoke vs. evacuating from an active fire), these possibilities warrant future research.

Beyond our unique sample of PCT hikers, individuals encounter climate change impacts when they are exposed to smoke from an active fire, are forced to evacuate their homes, experience a loss of personal property, or face uncertainty due to changing temperatures and weather patterns. Therefore, while our results are not generalizable beyond the specific PCT population, their implications may be transferable to other contexts in which individuals experience climate change impacts (Jahn and Myers, 2024; Morgan, 2007). However, more research is needed to confirm these relationships among individuals who are exposed to extreme wildfires in other contexts, and to examine the ways in which other extreme weather events relate to the hypothesized model.

### 6.2 Implications for climate change communication

These results suggest several implications for climate change communication. Wildfires and extreme weather events are accompanied by increased media attention during and immediately following the event (Crow et al., 2017; Johnson et al., 2009). Although media producers are increasing the frequency with which they mention climate change-related issues during their reporting of extreme weather (Hopke, 2020), there is still a long way to go (Cordner and Schwartz, 2019; Crow et al., 2017; Spialek et al., 2021). Furthermore, the media’s normative framing of behavioral responses to extreme weather can influence citizen responses (Nilsson and Enander, 2020), but most do not provide information on risk mitigation, policy solutions, or other actions individuals can take in response to the event (Crow et al., 2017). This study’s preliminary findings suggest that not only should messaging during extreme weather events describe aspects of the event, but that highlighting the proximity (whether physical, temporal, social, or hypothetical) of climate change and relevant (favorable) normative standards around PEB may increase the behavioral impact of the exposure. If the results found within this sample of PCT hikers reflect processes that occur outside of this unique context, they may help media producers tailor their messaging on wildfire and extreme weather to motivate greater pro-environmental behavior. This messaging may be used in combination with other pro-environmental policy mixes to offset negative effects of single interventions (e.g., negative spillover; Alt et al., 2024). See Ettinger et al. (2023) for a framework for using extreme weather events as teachable moments to increase PEB.

Finally, this study contributes to an understanding of why exposure to wildfires may *not* lead to changes in psychological proximity, attitudes, and PEB (Maiella et al., 2020; Wang et al., 2022). Although not hypothesized, the semi-structured interview guide allowed participants to expand upon this lack of association, and they mentioned several barriers to these associations. The most frequent included a lack of efficacy as a result of understanding the scope of environmental challenges. For example, many described the sense of helplessness that came from watching such a catastrophic impact, with one noting that:

I feel like there are two general ways that you can react to very present examples of climate change like fire. Some people, I think, get very invigorated by that and become very active and have decided this is a time when I choose what I want to be. For myself, I actually find that it’s the cause of a lot of apathy. The scope of things is so incredibly overwhelming for me that it’s hard for me to believe that my actions will make a difference (4).

This apathy was often accompanied by an argument that individual actions are not enough, such as one hiker’s description that “My individual choices are not gonna mean anything if Amazon keeps overnight packages everywhere. I do not know that the wildfires, necessarily, have changed that” (41). The lack of efficacy reflects studies of CLT that demonstrate that psychologically proximate events prompt individuals to think about an activity in terms of *how* they will perform an action, whereas psychologically distant events promote greater consideration of *why* they will act (Liberman et al., 2007; McCrea et al., 2008). When wildfires make the psychological presence of climate change feel more immediate, it is possible that reflecting on *how* one would contribute to climate change mitigation increases the salience of barriers to PEB. If this is the case, individuals exposed to wildfires and other climate change impacts may benefit from information that increases their perceived efficacy to perform PEB.

Other hikers anticipated dissipating urgency once they were removed from the wildfire impacts—a phenomenon that is likely to occur outside of the PCT community as well (Konisky et al., 2016). For example, one hiker acknowledged that his motivation to engage in PEB “might change for a few months, but then I’ll easily slip into the comfy life of just never worrying and not really caring” (32). In developing messages and other interventions to promote PEB during extreme weather events, barriers such as these need to be addressed before meaningful change can be expected (Steg and Vlek, 2009). Thus, a more expansive underlying model based on the theory of planned behavior (Ajzen, 1991) which explicitly includes the concept of perceived behavioral control, could motivate greater consideration of these and other barriers in influencing PEB.

### 6.3 Limitations

This study has several limitations. First, PCT hikers do not represent the general adult population (e.g., partially due to their extensive outdoor recreation; Andre et al., 2017; Baird et al., 2020; Cole, 2018; Wilcer et al., 2018). The respondents are long-distance hikers who, during their hike, may be confronted with significant forms of environmental change. Further, this population also likely encounters a ceiling effect whereby PCT hikers’ attitudes and PEB may already be high (noted in some of the interviews), which could have reduced the associations of wildfire exposure with attitudes and PEB reflected in the comments. However, these PCT hikers had experienced different levels of exposure to wildfires, making them particularly unique but also salient respondents, filling a gap in the literature. Thus this sample is an intentional, purposeful sample, reflecting the construal level theory proposition that psychological distance is more strongly affected by near-immediate and salient experiences. Thus there is no presumption of the sample or results representing more general populations. Second, even among the PCT hiker population, only hikers who were interested in and had time to participate in the interviews were included, which further limits the results. Therefore, the sample has ecological validity, and is purposive, but is a small, non-representative, convenience sample.

Third, coded content can only refer to reported or intended general propensities to engage in PEBs (Lange, 2024) as reflected in responses to the questions and in Figure 2, as there are no measures of observed PEB. However, a much larger sample could use the quantitative counts of each code to statistically test portions of, or the full, model. Fourth, we did not code for all constructs of the TRA as outlined in the theoretical model, so future research may explore more specific attitudes toward the behavior, and the beliefs underlying these behavior-specific attitudes. However, the codes and relationships in this study do represent a common adaptation of the TRA within our theoretically-justified model, and the most salient aspects of relevant participants’ experiences and perceptions. Finally, of course, with all *a priori* content analysis approaches, other potentially relevant comments about wildfire experiences and PEB that may have emerged through open coding were not represented.

## 7 Conclusion

This study examined how Pacific Crest Trail hikers’ wildfire exposure shapes their perceptions of climate change and pro-environmental behavior, using a model integrating construal level theory and the theory of reasoned action. A content analysis of 66 interviews identified that:

- Wildfire exposure reduced psychological distance to climate change across all four dimensions, and reinforced concern about both climate change and wildfires.
- Attitudes were more consistently linked to general PEB than to wildfire-specific PEB.
- Subjective norms—especially from fellow hikers—influenced general PEB but had limited influence on wildfire PEB.
- Despite greater perceived proximity, many participants described barriers (e.g., low efficacy, apathy) that limited behavior change.

Although our sample was small and not representative of the general population, studying the relationships between exposure to climate change impacts, psychological distance, attitudes, and PEB in real time clarifies mixed evidence in the literature and suggests opportunities for increasing environmental action. Future research can expand on these findings by studying broader populations, incorporating measures of perceived efficacy, and testing interventions that highlight proximity and social norms to promote climate action.

## Data Availability

The raw data supporting the conclusions of this article will be made available by the authors, without undue reservation.
